# Recent sequencing and phylogenetic analysis of equine herpesviruses 1 and 4 among different equine populations in Egypt

**DOI:** 10.5455/javar.2023.j719

**Published:** 2023-12-31

**Authors:** Mohamed El-Zayat, Omayma A. Shemies, Samah M. Mosad, Sahar Abd El Rahman

**Affiliations:** 1 Virology Research Department (VRD), Agricultural Research Center (ARC), Animal Health Research Institute (AHRI), Giza, Egypt; 2 Department of Virology, Faculty of Veterinary Medicine, Mansoura University, Mansoura, Egypt

**Keywords:** EHV-1, EHV-4, equine herpesviruses, equine industry, respiratory disease, virus identity

## Abstract

**Objective:**

Equine herpes viruses (EHVs) are considered one of the most important respiratory pathogens in equids, resulting in serious outcomes for equine health worldwide. The objectives of the current research were the detection, molecular characterization, and isolation of EHV-1 and EHV-4 circulating within different equine populations in Egypt, either clinically or in apparently healthy horses.

**Material and Methods:**

A total of 120 field samples were collected, and DNA was extracted. Screening and typing of extracted DNA were done by consensus and conventional PCR assays for detection of EHV-1 and EHV-4, followed by sequencing and phylogenetic analysis to confirm the virus identity. Selected positive samples for both EHV-1 and EHV-4 were subjected to Madin-Darby bovine kidney (MDBK) cell lines for virus isolation.

**Results:**

The obtained results revealed that 58/120 (48%) samples were positive for EHVs. Typing of positive samples showed that EHV-1 was detected in (48/120) 40% of samples and EHV-4 was detected in (15/120) 12% of samples, while dual infection by both EHV-1 and 4 was detected in five samples.

**Conclusion:**

The current study revealed new data on the continuous circulation of EHV-1 and EHV-4 within equine populations in Egypt, and individual horses could be infected by multiple EHVs. In addition, latently infected horses are acting as potential reservoirs for frequent virus reactivation.

## Introduction

Equine herpesviruses (EHVs) are considered one of the most devastating threats to equine health globally, resulting in great economic losses and welfare consequences for the equine industry [1]. Infection by EHVs in horses takes several forms, including abortion in pregnant mares**,** neonatal fetal mortality, acute upper respiratory tract disease, and ocular and neurological disorders [2].

EHV-1 and EHV-4 are the most important EHVs responsible for the infection in most horses worldwide, resulting in respiratory manifestations [3]. Infection by EHV1 causes abortion in pregnant mares, perinatal mortality, and myelencephalopathy [4,5]**,** while EHV-4 infections cause respiratory disorders and occasionally abortion [6].

EHVs are a group of viruses within the family Herpesviridae, including nine EHVs from EHV1 to EHV9. Until now, six of them (EHV-1, EHV-3, EHV-4, EHV-6, EHV-8, and EHV-9) belong to the subfamilies Alphaherpesvirinae, while the other three viruses (EHV-2, EHV-5, and EHV-7) belong to Gammaherpesvirinae. Horses have been infected by five subtypes of equine herpesviruses (from EHV1 to EHV5), while donkeys are the natural hosts of EHV6, EHV-7, and EHV8 (also referred to as asinine herpesviruses AHV1, AHV-2, and AHV3, respectively). Equine herpesvirus EHV9 (gazelle herpesvirus) has been detected in Thomson’s gazelles, giraffes, and zebras [7–9].

One particular aspect of EHVs is the establishment of life-long latent infection, which is considered a challenge to controlling virus transmission as clinical manifestations are difficult to identify during the process of reactivation, and latently infected horses are considered a source of infection [10,11].

Definitive laboratory diagnostic techniques for the detection of EHVs have been developed involving viral isolation and serological assays, including virus neutralization tests and enzyme-linked immunosorbent assay (ELISA) [3]. Recently, different assays of PCR have been developed for molecular detection and typing of EHVs through the use of specific primers and probes [12–14].

In Egypt, monitoring of EHVs is sporadic, and different EHVs have been detected from clinical samples collected from horses, but the available studies about EHVs (including sequence and phylogenetic analysis) are not enough to describe the epidemiological state of EHVs as documented by different authors in previous studies, and most of these studies focused on the prevalence and isolation of EHVs. In addition, scanty studies are available concerning EHV-4 and its role during abortion outbreaks [15–22].

Therefore, the objectives of the current study were monitoring, sequencing, phylogenetic analysis, and isolation of EHV-1 and EHV-4 circulating in Egypt within different equine populations (either diseases or apparently healthy horses) using the most recent molecular diagnostic techniques.

## Materials and Methods

### Ethical statement

All sampling and examination procedures were performed with the permission of the Faculty of Veterinary Medicine, Mansoura University, Egypt, which complies with all legal requirements in Egypt with approval number Ph.D./45/2019.

### Samples collection

A total of 120 samples were collected from different equine populations, including studs and equine clinics, in different localities in Egypt (Cairo, Giza, Dakahlia, Sharkia, and Gharbia Governorates) (Table 1). Nasal swabs (*n* = 62) were taken from horses suffering from respiratory manifestations using sterile cotton swabs. Uterine swabs (*n* = 20) and tissue samples (*n* = 10) (from aborted fetuses and placenta) were obtained from aborted mares. Whole blood samples (*n* = 28) were collected from apparently healthy mares with a previous history of abortion. Samples were placed directly in transport media and transported immediately in coolers to the laboratory for sample preparation and further examination.

### Viral DNA extraction

DNA was extracted from prepared field samples using the commercial kit QIAamp^®^ DNA Mini Kit (Cat. No. 51304) or QIAamp^®^ DNA Blood Mini Kit (Cat. No. 51104, purchased from Qiagen GmbH, Hilden, Germany) according to the kit’s instructions using QIAamp Mini Spin Columns for DNA extraction and finally eluted in 50 μl elution buffer.

**Table 1.** Details of collected samples from different equine farms and clinics.

Stud, clinic ID	History	Type and no. of samples	Location
Stud A	Respiratory signs	Nasal swabs/15	Cairo
	Abortion	Uterine swabs/5 Tissue/10	
	No obvious clinical signs	Whole blood/28	
Clinic A	Respiratory signs	Nasal swabs/10	
Stud B	Respiratory signs	Nasal swabs/6	Giza
	Abortion	uterine swabs/4	
Clinic B	Respiratory signs	Nasal swabs/10	Dakahlia
Stud C	Respiratory signs	Nasal swabs/12	Sharkia
	Abortion	uterine swabs/6	
Stud D	Respiratory signs	Nasal swabs/9	Gharbia
	Abortion	uterine swabs/5	

### Consensus herpesvirus PCR

Screening of EHVs in the extracted DNA was done by consensus PCR using a pan-EHV primer targeting the DNA polymerase gene, which amplifies 250 bp fragments. The first round includes two forward primers (DFA, 5'-GAY TTY GCN AGY YTN TAY CC-3'; and ILK, 5'-TCC TGG ACA AGC AGC ARN YSG CNM TNA A-3') and a single reverse primer (KG1, 5'-GTC TTG CTC ACC AGN TCN ACN CCY TT-3'). The second round of PCR was done on 5 ml of the first PCR mixture in a 50 ml volume with forward primer TGV (5'-TGT AAC TCG GTG TAY GGN TTY CAN GGN GT-3') and reverse primer IYG (5'-CAC AGA GTC CGT RTC NCC RTA DAT-3'). The reactions were done for 45 cycles of denaturation for 30 sec at 94°C, annealing for 60 sec at 46°C, and extension for 60 sec at 72°C. The final extension was conducted for 7 min at 72°C [23].

### Conventional PCR assay

Typing of positive samples for detection of EHV-1 or EHV-4 was performed by conventional PCR using primers targeting glycoprotein B (gB) (Table 2). A total of 25 μl of the mixture was prepared: 12.5 μl of 2x Emerald Amp GT PCR Master Mix (TAKARA-BIO, Japan), 1 µl of forward primer, 1 µl of reverse primer, and 5 μl of extracted DNA. The reaction was completed in 25 μl using nuclease-free water. The amplification was done in a thermocycler (Biometra, Germany). The thermal profile for EHV-1 was performed as follows: 35 cycles (denaturation for 45 sec at 95°C, annealing for 45 sec at 55°C, extension for 60 sec at 72°C), and final extension for 10 min at 72°C. While PCR reactions for EHV-4 were done as follows: 35 cycles (denaturation for 60 sec at 94°C, annealing for 60 sec at 66°C, extension for 60 sec at 72°C, and final extension for 10 min at 72°C).

**Table 2.** List of Primers used in conventional PCR.

Target gene	Primer	Primer sequence (5’–3’)	Product size	Ref.
EHV-1(gB)	For Rev	CACTTCCATGTCAACGCACT TCGACTTTCTTCTCGGTCCA	869 bp	[20]
EHV-4(gB)	For Rev	TATTGTTTCCGCCACTCTTGACG GTAGAATCGGAGGGCGTGAAGC	508 bp	[24]

### DNA sequencing and phylogenetic analysis

The sharpest bands of conventional PCR products (gB genes) were selected for DNA sequencing. Selected bands were excised from the gel, purified using the QiAquick gel extraction kit (Qiagen, Germany), and sequenced by Applied Biosystems (ABI, Foster City, CA). Geneious software version 2020.1 was used for trimming vector contamination and low-quality reads. The obtained sequences were submitted to GenBank and compared to other EHV isolates published in the Genbank database by using the basic local alignment search tool (BLAST) of the National Center for Biotechnology Information (NCBI) for detection of sequence similarity. The obtained sequences were aligned through clustal W alignment, and a neighbor-joining tree with statistical analysis of 1000 replicates was constructed using MEGA 11 software. Nucleotide and deduced amino acid sequences were analyzed using BioEdit software [25].

### Isolation of *Madin-Darby bovine kidney* (MDBK) cells

MDBK cell lines were examined for the quality of the cells, trypsinized with 0.25% trypsin supplemented with 2.5 μmol·L^-1 EDTA^, and then growth media (minimal essential media (MEM containing 10% new-born calf serum and twice the standard concentrations of antibiotics (200 U ml^-1^ penicillin and 200 μg ml^-1^ streptomycin) was added to the cell suspension. The suspension was distributed into disposable plastic prescription flasks (T 25) and incubated at 37°C with 5% CO_2_ till the cells formed a 60%–80% monolayer confluent sheet. Molecularly identified positive samples representing EHV-1 and EHV-4 were selected, and 100 μl from each sample was inoculated.

## Results

### Molecular screening and typing of EHV

Screening of EHV in the extracted DNA by consensus PCR revealed that 58/120 (48%) samples were positive for EHVs. Typing of positive samples by conventional PCR showed that EHV-1 was detected in (48/120) 40% of samples and EHV-4 was detected in (15/120) 12% of samples (Table 3), while dual infection by both EHV-1 and 4 was detected in five samples.

**Table 3.** Presence of EHVs in samples detected by both consensus and conventional PCR.

Sample type	No. of samples	No. of positive samples	EHV-1	EHV-4
Nasal swabs	62	25	21	7
Uterine swabs	20	10	8	2
Tissue	10	5	4	1
Whole blood	28	18	15	5
Total (%)	120(100%)	58(48%)	48(40%)	15(12%)

### Sequencing and phylogenetic analysis

**Figure 1.**
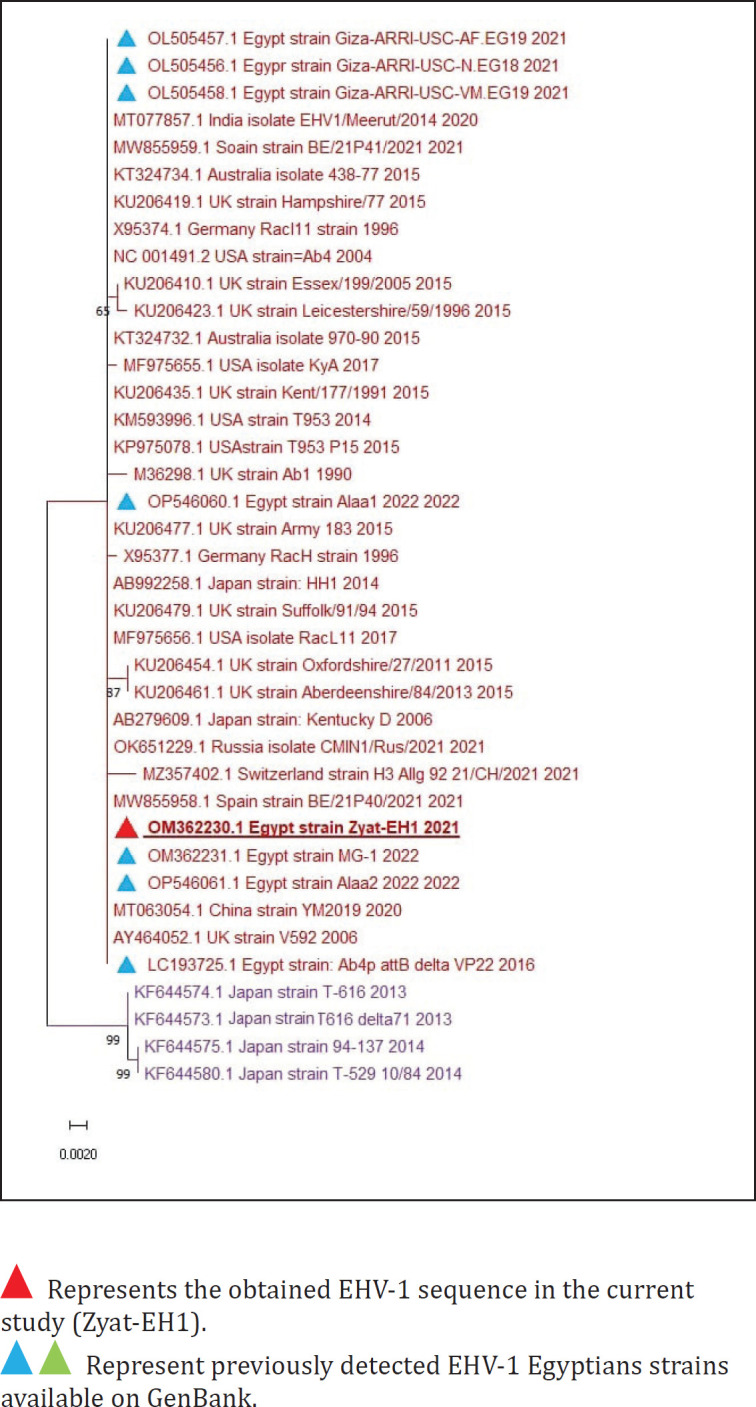
Phylogenetic tree of EHV-1.

Sequenced samples were analyzed, the sequences were submitted to GenBank, and the accession numbers were obtained as follows: OM362230 for the obtained EHV-1 sequence (strain Zyat-EH1) and OM362233 for the obtained EHV-4 sequence (strain Zyat-EH4). Phylogenetic analysis of the obtained sequence of EHV-1 (strain Zyat-EH1) with other reference EHV-1 sequences from GenBank revealed that the phylogenetic tree was divided into two clusters. All Egyptian EHV-1 strains (including the Zyat-EH1 strain) were aligned in the first cluster, while the second cluster included EHV-1 strains from nonequine hosts: T616 (Zebra), T-529 (Onager), and strain 94–137 (Gazelle) (Fig. 1). The Zyat-EH1-EH1ain showed 100% identity with all other Egyptian strains, while the Zyat-EH1 strain showed 98.26%–100% identity with other reference EHV-1 strains from other countries.

**Figure 2.**
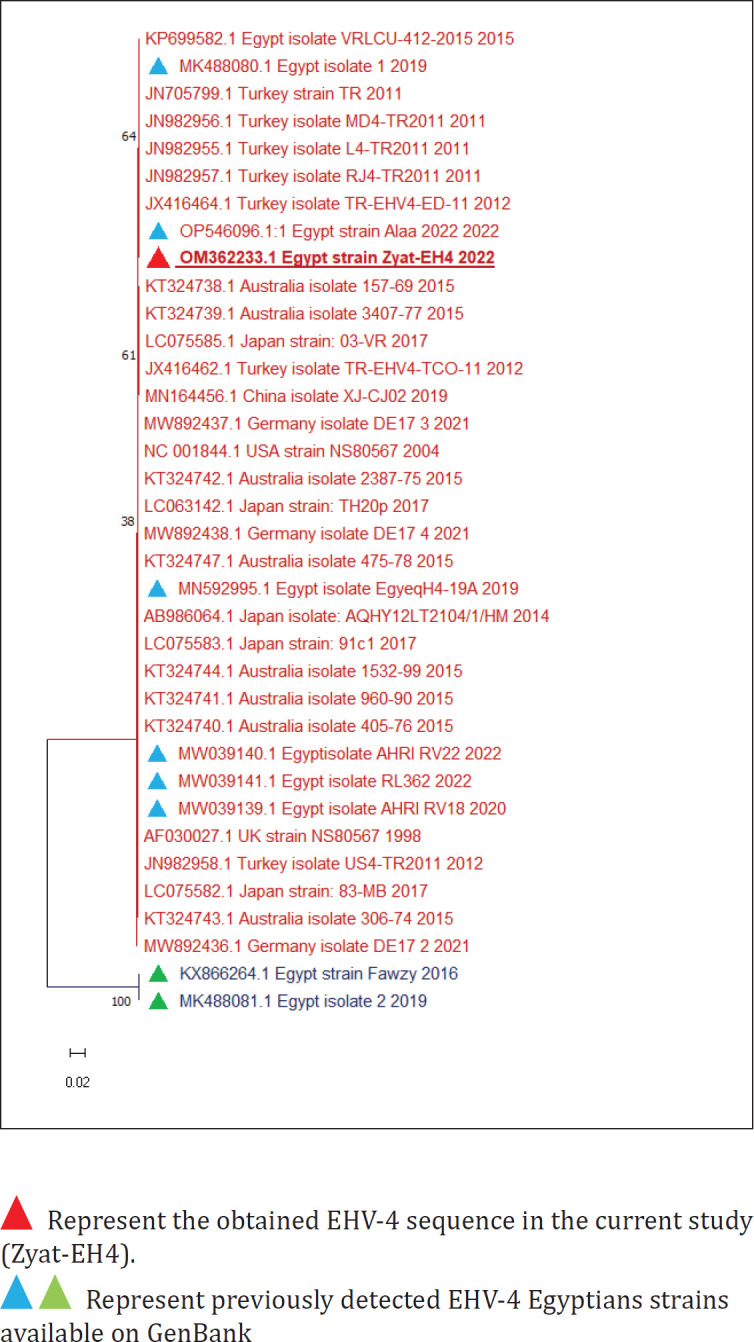
Phylogenetic tree of EHV-4.

The obtained EHV-4 (Zyat-EHV4) sequence was aligned with previously identified EHV-4 Egyptian strains and other foreign strains from GenBank, and the phylogenetic tree was divided into two clusters (Fig. 2). Zyat-EHV4 was aligned in the first cluster along with Egyptian EHV-4 strains (with identity percent 99.6%–100%) except for the Fawzy_2016 and strain isolate_2_2019 Egyptian strains, which formed a distinct cluster with identity percent 75.38% and 75.45%, respectively. Zyat-EHV4 was identical to the Egyptian strains (Alaa-2022, VRLCU-412, and strain 1_2019) and similar to the Egyptian strains EgyeqH4-19A, AHRI_RV22, RL362, and AHRI_RV18 with 99.76% identity.

Nucleotide and deduced amino acid sequence analysis

Nucleotide analysis of the obtained EHV-1 sequence with horse-derived EHV-1 strains showed none or few nucleotide substitutions (1–3 nucleotides), while the obtained EHV-1 sequence was differentiated from EHV-1 strains isolated from different hosts other than horses (Zebra, Onager, and Gazelle) by 14–15 nucleotides that led to only a single amino acid substitution.

Analysis of the obtained EHV-4 sequence revealed 0–2 nucleotide substitutions (that led to none or single amino acid substitution) with other EHV-4 foreign and Egyptian strains except strains Egypt_strain_Fawzy_2016 and Egypt_isolate_2_2019. The nucleotide substitutions were 112 and 108, respectively, which led to only a single amino acid substitution in both strains.

### Isolation in MDBK

**Figure 3.**
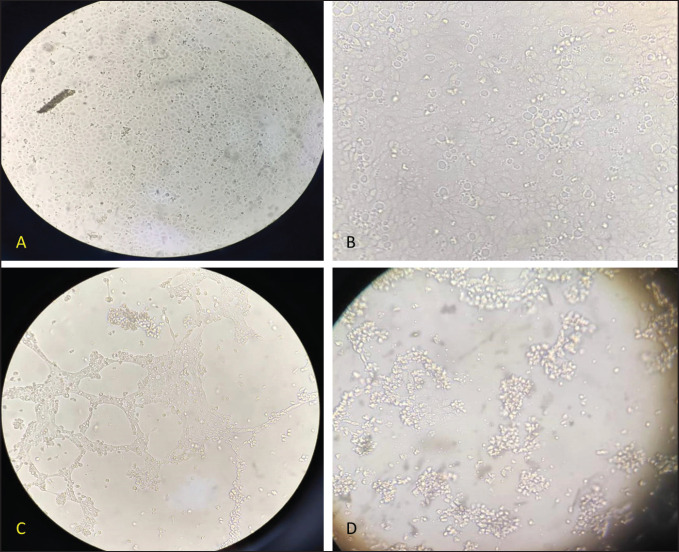
Cytopathic effect in MDBK cells. (A) Negative control (confluent monolayer cells). (B–D) CPE in MDBK cells.

Propagation of molecularly identified samples on MDBK cell culture for three successive blind passages. Control prescriptions show confluent monolayer cells (Fig. 3A). The cytopathic effect (CPE) appeared after 3 days from inoculation and was recorded for each sample as an aggregation of cells, focal rounding, and finally cells detached (Figs. 3B–D). After 5 days, freezing and thawing were done three times for the harvesting of the propagated virion.

## Discussion

In Egypt, monitoring of EHVs is sporadic, but infections by different types of EHVs have been detected in different horse populations, causing great economic losses that have reached millions of Egyptian pounds, as the Arabian horse breed is highly valuable among equine breeds used for export, showcasing, and semen collection [15,^16^,20].

The current research was conducted for the detection, molecular characterization, and isolation of EHV-1 and EHV-4 circulating within different horse populations, including studs or clinics in different localities in Egypt, using the most recent molecular diagnostic techniques to achieve a better understanding of the epidemiological status of EHVs and the application of a robust control program.

A total of 120 samples were obtained from either apparently healthy or clinically diseased horses suffering from respiratory manifestations or abortions suspected to be infected by EHVs. EHV-1 and EHV-4 are still circulating within different equine populations. EHV-1 was detected in 40% of the collected samples, revealing that EHV-1 is still the predominant EHV responsible for respiratory manifestation and abortion in horses, resulting in great health consequences and economic losses as described by [26,27]. Screening and typing of uterine swabs and tissue samples collected from aborted mares revealed that EHV-1 was responsible for most abortion cases in mares that mostly occurred at 6–9 months of gestation, which coincided with previous reports [2,28].

EHV-4 was detected in 12% of the collected samples, resulting in an increase in the susceptibility threshold to infection by EHV-4 owing to the fact that the current local vaccine used in Egypt contains only the EHV-1 strain. EHV-4 was identified in the abortion cases, confirming that EHV-4 contributed to the abortion outbreaks in pregnant women, which coincides with previous studies [16,^19^,^29^,30]. EHV-4 was detected individually in two uterine swabs and one tissue sample, which confirmed that EHV-4 is still incriminated as a primary cause of abortions, which is in harmony with previous studies by Al-Shammari et al. [29] and Khattab et al. [31]. In addition, EHV-4 was detected in nasal swabs, the primary known samples, that agreed with EHV-4 pathogenesis as described by previous studies [16,19].

Dual infection by both EHV-1 and EHV-4 was recorded, which confirmed that individual horses could be infected by multiple EHVs, which agreed with prior reports [20,24] revealing that it is highly recommended to develop preliminary molecular assays for screening of EHV-1 and 4, particularly during abortion outbreaks since the two viruses have been detected within the same sample.

Screening results of whole blood samples collected from apparently healthy mares with a previous history of EHV infection confirmed the highly advanced immune evasion strategies of EHVs that remain latent in infected horses for a long time. Virus reactivation from latently infected horses under stress conditions is considered a continuous threat for equine populations, revealing that stress factors are crucial during the outbreak of the disease [32]. It is worth mentioning that different EHVs could be detected from blood samples collected from apparently healthy horses [20,24].

A PCR assay targeting a highly conserved region of gB plays a valuable role in the typing of EHVs in the collected samples since it is highly sensitive and specific for the detection of EHV nucleic acid to clarify the circulation and epidemiological investigation of EHVs within different equine populations, as previously documented [16,20–29]. Moreover, the sensitivity of PCR assays overcomes the negative results obtained by serological methods in either latently or recently infected horses.

The phylogenetic analysis of the EHV-1 sequence in the current study was homogenous with the previously detected Egyptian EHV-1 strains and aligned in one cluster, showing complete identity, while the obtained EHV-1 sequence was similar to other reference EHV-1 strains from other countries, indicating that there is very little nucleotide substitution, very stable genomic DNA of EHVs, and no diversity in the gB gene as reported by previous studies [33,^34^,35].

While the phylogenetic analysis of the EHV-4 sequence was similar to the previously detected Egyptian strains available on GenBank except for strains Egypt fawzy_2016 and Egypt isolate_2_2019, despite the fact that there were many nucleotide substitutions in both sequences, only a single amino acid substitution was found. Therefore, it is advised to conduct further in-depth analysis by EHV-4 whole genome sequencing during outbreaks of abortion to reveal any amino acid substitutions that could modify the viral pathogenesis and result in a change in the behavior of the virus.

Furthermore, the findings of phylogenetic analysis of the obtained EHV-1 and EHV-4 sequences in the current research showed that they were similar to foreign strains, which may be due to the continuous crossing of borders to participate in global races or showcases that increase the incidence of infections by foreign EHVs. Therefore, it is highly recommended to examine all horses, either imported horses or horses that participated in races upon return against EHVs.

Tissue culture is still the gold standard technique for virus isolation, so the molecularly identified field samples were selected and successfully propagated on MDBK cell lines for three blind passages and displayed positive cytopathological changes such as aggregation of cells, focal rounding, and finally, cells detached. These findings coincide with those described by the OIE [36].

## Conclusion

In conclusion, it is highly recommended to establish reliable monitoring procedures for the detection of different EHVs and preventive measures to control the disease in Egypt, and it is also necessary to update the current local vaccine for developing efficient EHV vaccines that protect the equine population against EHVs. In addition, further molecular and epidemiological investigation is required, especially for EHV-4, to reveal any alteration in the known viral pathogenesis, as EHV-4 was previously known as a respiratory pathogen, but recently it has a significant role in abortions that needs more investigation.

## List of abbreviations

DNA, deoxy ribose nucleic acid; PCR, polymerase chain reaction; EHVs, Equine herpesviruses; MDBK, Madin-Darby bovine kidney; CPE, cytopathic effect; MEM, minimal essential media; ELISA, enzyme-linked immunosorbent assay; gB, glycoprotein B.
